# Assessing the intestinal carriage rates of vancomycin-resistant enterococci (VRE) at a tertiary care hospital in Hungary

**DOI:** 10.1007/s12223-019-00751-x

**Published:** 2019-11-04

**Authors:** Dorottya Franyó, Balázs Kocsi, Evelin Erzsébet Bukta, Judit Szabó, Zsuzsanna Dombrádi

**Affiliations:** 1 Department of Medical Microbiology, Faculty of Medicine, University of Debrecen, Nagyerdei krt. 98, Debrecen, 4032 Hungary; 2 Institute of Industrial Process Management, Faculty of Engineering, University of Debrecen, Debrecen, Hungary

**Keywords:** VRE carriage, Risk factors, Antibiotic resistance, PCR, MALDI-TOF

## Abstract

Excessive use of antibiotics contributes to the selection of resistant bacteria and intestinal colonization with multiresistant pathogens poses a risk factor for subsequent infections. The present study assessed vancomycin-resistant enterococci (VRE) carriage rates in patients admitted to our tertiary care hospital. Stool samples sent for routine culturing were screened with vancomycin containing solid or broth enrichment media. VRE isolates were identified with matrix-assisted laser desorption/ionization-time of flight mass spectrometry and antibiotic susceptibilities were tested by E-test. Vancomycin resistance genes were detected by polymerase chain reaction. Medical records of carriers were examined for suspected risk factors for colonization. Altogether 3025 stool specimens were analyzed. Solid media identified a VRE carriage rate of 2.2% while broth enrichment detected 5.8%. Seventy percent of the isolates were *Enterococcus faecium. VanB* genotype was detected in 38.2%, *VanA* in 37.3%, *VanC1* in 22.6%, and *VanC2* in 1.9%. All VRE were sensitive to linezolid, daptomycin, and tigecycline. Collective risk factors for carriage were diabetes, normal flora absence, *Clostridioides difficile* positivity, longer hospital stay, and advanced age. 78.5% of the carriers received antibiotic therapy which was metronidazole in most cases (47.3%)*.* We recommend regular screening of risk groups such as patients with diabetes, history of recent hospitalization, or former *C. difficile* infection as an imperative step for preventing VRE dissemination.

## Introduction

Vancomycin-resistant enterococci (VRE) have emerged as one of the leading causes of multidrug-resistant hospital-acquired infections since their first isolation in the UK and France in 1986 (Uttley et al. 1988; Leclercq et al. 1988). According to the latest report of the European Centre for Disease Prevention and Control enterococci, VRE were the second most common bacteria causing bloodstream and urinary tract infections among hospital-acquired infections in intensive care units with a vancomycin resistance rate of 2.4% (ECDC 2016). The percentages of vancomycin-resistant *Enterococcus faecium* (VREfm) isolates in Europe ranged from 0% (Estonia, Finland, Iceland, Luxembourg, and Slovenia) to 46.3% (Cyprus) in 2016. VREfm isolate proportion increased markedly in Denmark (4.3–7.5%), Italy (4.4–13.4%), Bulgaria (2.3–18.2%) Croatia (6.8–22.1%), Slovakia (7.6–26.4%), and Romania (11.1–39%) between 2013 and 2016 (ECDC 2017). A rising trend can be also seen in Hungary in the same time period (7.1–22.4%). In accordance with the international and national trends, we recently reported the elevation of clinical VREfm from 1.7 to 11% (2012–2015) at our tertiary care hospital in Debrecen, Eastern Hungary (Franyó et al. 2018).

Enterococci are ubiquitous in gastrointestinal tracts even though they constitute a small proportion approx. 0.01% of the normal bowel flora (Jett et al. 1994). Typical concentrations in stool are up to 10^8^ CFU/g in humans. The oral cavity and vagina can also become colonized but enterococci are recovered from these sites in fewer than 20% of the cases (Huycke et al. 1998). Inadequate antibiotic administration is one of the main reasons for selection of resistant bacteria and long-term carriage of multiresistant pathogens increases the probability of subsequent infections. A number of risk factors have been associated with VRE colonization. These include prolonged hospital stay, advanced age, severe underlying disease, central venous catheterization, and exposure to various antibiotics such as vancomycin or metronidazole (Karki et al. 2012; Purohit et al. 2017; Sohn et al. 2013). Asymptomatic carriage of VRE and the lack of effective decolonization regimen stabilize their endemicity in the healthcare settings (Cheng et al. 2014). This colonization may serve as a reservoir for the transmission to other patients resulting in increased morbidity, mortality, and cost (Muto et al. 2003; Stosor et al. 1998).

In the present study, we aimed to determine the rate of VRE intestinal colonization among the patient population at our 1600-bed hospital. Previous surveys have demonstrated various colonization rates which were largely influenced by the sample types (rectal swab or stool sample) and by the culturing methods (direct plating on solid screening medium or broth enrichment) (D’Agata et al. 2001; Ieven et al. 1999). Therefore, we investigated the solid screening method and also the broth enrichment for screening VRE. We used matrix-assisted laser desorption/ionization-time of flight mass spectrometry (MALDI-TOF MS) for the species identification of our fecal isolates. Besides phenotypic and genotypic characterization, we also investigated various risk factors which could be associated with VRE colonization.

## Methods

### Sample collection and study period

This retrospective study was conducted in our tertiary care 1600-bed University Hospital in Debrecen, Hungary. We tested a total of 3025 stool specimens which were submitted to the microbiology laboratory for routine culturing between 2016 February and September. The examination period was divided into two phases. In the 1st period (155 days, 2050 specimens), stools were inoculated with a glass stick onto solid VRE screening medium containing 6 μg/mL vancomycin (VRE agar base, Oxoid). In the 2nd period (88 days, 971 specimens), the same inoculation technique and culture medium was used but without solidification with agar (broth enrichment). After 24–48 hours of incubation at 37 °C, black colonies from the agar or broth tubes that turned black were subcultured on 5% blood agar (LabM) and stored in Tryptone Soy Broth with glycerol (LabM) at − 20 °C until further testing. Multiple stools from the same patient were counted as a single result if all yielded species and their characteristics were indistinguishable.

### Species identification with MALDI-TOF mass spectrometry

Enterococcal isolates were identified to the species level by MALDI-TOF MS using a Microflex LT/SH instrument (Bruker Daltonics). The formic acid extraction method was used according to the manufacturer’s instructions and protein peaks were detected in the 2000–20,000 Da mass range. Each sample was tested twice by measuring 2 spots on the target plate. The MALDI Biotyper 3.0 software with the integrated database containing 5627 spectra was used for species determination. Isolates showing score values > 2.0 were accepted.

### Antimicrobial susceptibility testing

Vancomycin, teicoplanin, daptomycin, linezolid, tigecyline, and quinupristin/dalfopristin minimum inhibitory concentrations (MICs) were determined by E-test strips (BioMerieux) according to the manufacturer’s instructions. The European Committee on Antimicrobial Susceptibility Testing guidelines were applied to interpret the results (EUCAST 2018). ATCC 29212 *E. faecium* was used as the quality control strain.

### Molecular detection of glycopeptide resistance

Lysates of bacteria were prepared by the boiling method of Yean et al. (2007). Polymerase chain reactions (PCR) were performed in an S1000 thermal cycler (Bio-Rad). Conditions and primers for amplification of the *VanA*, *VanB*, and *VanC1/C2* resistance genes were used as described previously (Depardieu et al. 2004).

### Epidemiological data

Medical records of patients with VRE-positive fecal samples were investigated 6 months prior screening for the following factors: age, sex, underlying disease, length of hospitalization, antibiotic consumption, intravenous catheter, dialysis, nasogastric tube, tracheostomy, ventilator use, absence of normal enteric flora, and presence of toxin positive *Clostridioides difficile*. We also analyzed our database for any other VRE-positive clinical specimen of the carriers 6 months preceding and following our screening. For statistical analysis, we also investigated 93 VRE negative fecal samples; patients in this population were selected by Monte-Carlo simulation based on random number generation. Medical data was evaluated with the SPSS software version 24.0 (SPSS Inc.). The outcome of colonization with VRE was expressed as binary categorical variable. All categorical variables were analyzed by chi-square test. If all expected value frequencies were not equal to or greater than 5, Fisher exact probability test was applied, while logistic regression was used for continuous independent variables and categorical outcome. *p* value less than 0.05 was considered statistically significant. Additionally, odds ratio was utilized to determine the association between risk factors and VRE colonization.

## Results

During the first research period, a total of 2050 samples were screened by solid screen. Three hundred thirteen samples (15%) yielded black colonies on the screen agar. Naturally, this is not equal to the carriage rate of VRE since other generically vancomycin-resistant species such as *Lactobacillus*, *Pediococcus*, or *Weisella* can also grow on the screen agar. Additionally some plates grew Gram-negative bacteria or fungi from the normal bowel flora. Therefore, only 266 isolates were selected based on their colony morphology on blood agar for MALDI-TOF measurements. In the second research period, 971 samples were screened with enrichment broth. Among them, 469 (48%) caused blackening of the broth and according to their macroscopic morphology, 264 isolates were selected for MALDI-TOF analysis. Two colonized patients had different VRE species isolated from consecutive samples and 7 yielded two different VRE species from the same sample. These were all considered independently positive so altogether a total of 102 non-duplicate VRE were obtained from 93 patients.

**Fig. 1:**
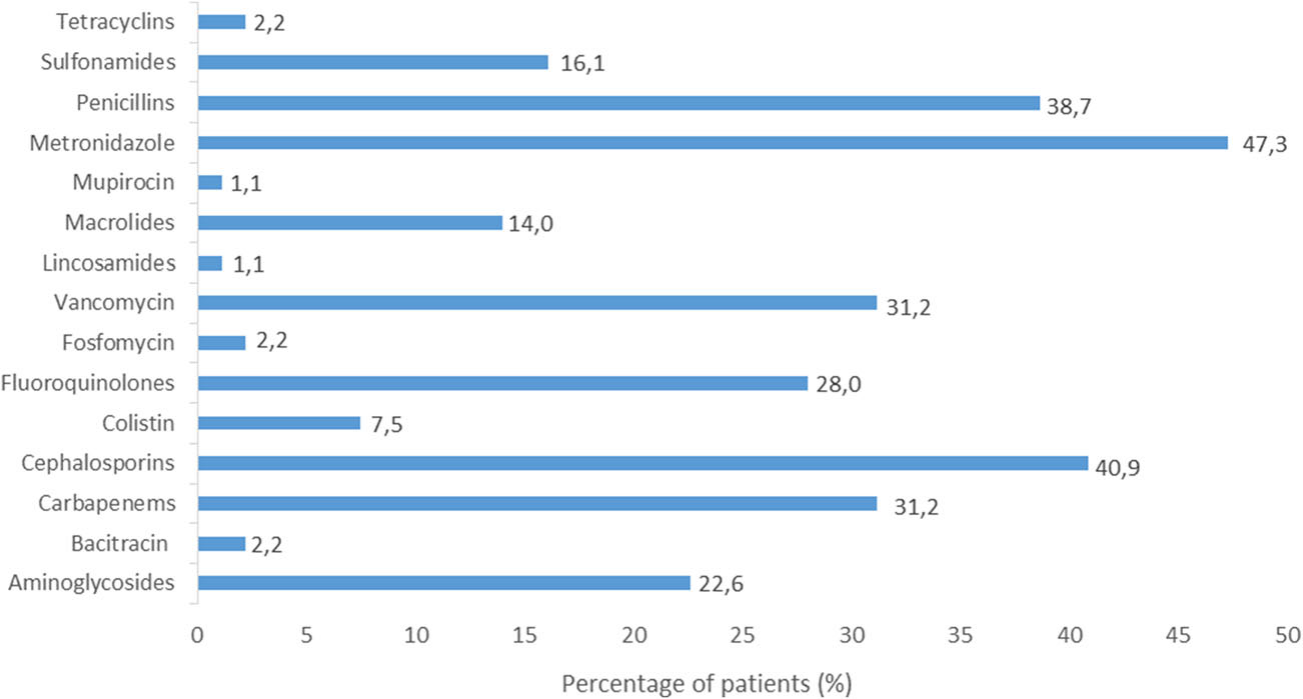
Antibiotic consumption in the period of 6 months prior screening

The characteristics of the patients are summarized in Table 1. VRE carrier’s age ranged from 8 months to 102 years with a mean of 47 years. Majority of them (71%) were adults and females were slightly predominant (59.1%). The mean length of hospital stay prior screening was 25 days. Only 7 carriers were outpatients. The most prevalent underlying disease was malignancy (22.6%) followed by diabetes and viral or bacterial infection (21.5%). Among the investigated risk factors, absence of normal enteric flora was found in 24.7%. Eighteen carriers (19.4%) had toxin positive *C. difficile* detected at least once at the time of screening or in the previous 6 months. Mechanical ventilator usage was also common (10.8%). Statistical analysis of 93 colonized and non-colonized patients showed that diabetes, normal flora absence, *C. difficile* positivity, longer hospital stay, and advanced age were significantly associated with VRE colonization. The highest chance occurred in the case of diabetes. The chance of VRE positivity was 12.46 times higher in these patients (Table 1). 78.5% of the VRE colonized patients received at least one kind of antibiotic during the 6 months prior stool sampling. Among them, metronidazole was the most commonly administered (47.3%) followed by cephalosporins (40.9%) (Fig. 1).

**Table 1:** Patient characteristics associated with VRE colonization

Patient characteristics	VRE colonization *n* = 93 (%)	VRE not present *n* = 93 (%)	OR (95%–CI)	*p* value
Age (years, mean ± SD)*	47 ± 34.2	29 ± 32.7	1.016 (1.007–1.025)	< .0001
Female sex	55 (59.1)	49 (52.7)	1.299 (0.727–2.321)	0.37
Hospital stay (days, mean, range)*	25, 0–215	11, 0–70	1.042 (1.024–1.060)	< .0001
Underlying disease				
Diabetes*	20 (21.5)	2 (2.2)	12.46 (2.822–55.08)	< .0001
Cardiovascular	9 (9.7)	6 (6.4)	1.55 (0.529–4.554)	0.420
Malignancy	21 (22.6)	13 (14)	1.79 (0.838–3.843)	0.129
Cirrhosis	2 (2.2)	2 (2.2)	1 (0.137–7.253 )	1
Pulmonary disorder	3 (3.2)	2 (2.2)	1.51 (0.247–9.293)	0.5
Gastrointestinal disorder	4 (4.3)	7 (7.5)	0.55 (0.156–1.953)	0.26
Infection^1^	20 (21.5)	58 (62.7)	0.16 (0.086–0.316)	< .0001
Kidney failure	7 (7.5)	1 (1.1)	0.133 (0.016–1.107)	0.032
Other^2^	7 (7.5)	2 (2.2)	0.27 (0.054–1.335)	0.08
Risk factors				
Intravenous catheter	5 (5.4)	14 (15)	0.32 (0.110–0.930)	0.029
Dialysis	3 (3.2)	1 (1.1)	3.06 (0.313–30.035)	0.31
Nasogastric tube	3 (3.2)	4 (4.3)	0.7417 (0.161–3.409)	0.5
Tracheostomy	6 (6.5)	1 (1.1)	6.34 (0.748–53.778)	0.059
Ventilator use	10 (10.8)	6 (6.4)	1.74 (0.607–5.021)	0.296
Normal flora absence*	23 (24.7)	4 (4.3)	7.31 (2.412–22.118)	0.0001
*C. difficile* positivity*	18 (19.4)	8 (8.6)	2.55 (1.048–6.202)	0.0344

*SD*, standard deviation

^1^Bronchitis, pneumonia, urinary tract infection, diarrhea, sepsis, viral infection, toxic shock syndrome, infant respiratory distress syndrome; ^2^hypovolemia, aplastic anemia, fluid imbalance, hepatosplenomegaly, hypogammaglobulinemia, systemic lupus erythematosus

*Significantly associated with VRE colonization

Identification with the mass spectrometer revealed that 71 VRE were *Enterococcus faecium*. Interestingly the second most common species was *E. gallinarum* (*n* = 23) followed by 5 *E. faecalis*, 2 *E. casseliflavus*, and 1 *E. hirae* (Table 2). Among the *E. faecium* isolates, 33 were *vanA* and 38 were *vanB* positive. Only 4 *vanA* and 1 *vanB* positive *E. faecalis* were detected. The single *E. hirae* was found to be *vanA* positive. Naturally all *E. gallinarum* carried the *VanC1* and *E. casseliflavus* the *VanC2* genes. *VanB* positive isolates showed high-level resistance against vancomycin (median MIC > 256 μg/mL) and sensitivity to teicoplanin whereas the *vanA* positive isolates displayed high-level resistance against both antibiotics (median vancomycin MIC > 256 μg/mL; median teicoplanin MIC = 32 μg/mL) (Table 2). All of the *vanC* strains had low-level vancomycin resistance (median MIC = 6 μg/mL) and were susceptible to teicoplanin. Fortunately, all isolates were susceptible to newer antibiotics such as linezolid, tigecycline, and daptomycin. Quinupristin/dalfopristin resistance was found only in 4 *E. faecalis* isolates (3.9%) (Table 2).

**Table 2:** Antibiotic resistance of fecal VRE isolates

Species no.	MIC range μg/mL (resistance %)					
	Van	Tei	Lin	Dap	Tig	Q/D
*E. faecium* (71)	6–256 (100)	0.125–256 (47.9)	0.5–1 (0)	0.5–2 (0)	0.032–0.125 (0)	0.25–4 (0)
*E. gallinarum* (23)	4–6 (100)	0.125–0.5 (0)	0.25–1 (0)	0.5–2 (0)	0.032–0.125 (0)	0.5–4 (0)
*E. faecalis* (5)	> 256 (100)	0.125–256 (80)	0.5–1 (0)	0.125–1 (0)	0.032–0.125 (0)	4–8 (80)
*E. casseliflavus* (2)	6 (100)	0.125 (0)	0.5–1 (0)	0.5–1 (0)	0.064 (0)	1–2 (0)
*E. hirae* (1)	> 256 (100)	32 (100)	1 (0)	2 (0)	0.032 (0)	4 (0)

*MIC*, minimum inhibitory concentration; *Van*, vancomycin; *Tei*, teicoplanin; *Lin*, linezolid; *Dap*, daptomycin; *Tig*, tigecycline; *Q/D*, quinupristin/dalfopristin

Results of VRE screening using solid screen and enrichment broth are presented in Table 3. In the first period of investigation, the solid screening medium detected 46 VRE (2.2%) from 2050 stool samples while in the second period broth enrichment identified 56 VRE (5.8%) from 971 stools. Interestingly, 1 vancomycin-resistant *E. hirae* was also identified in the second period.

**Table 3:** VRE species detected by solid screen or broth enrichment

Isolate (no.)	No. (%) of isolates detected	
	1st period solid screen	2nd period broth enrichment
Total	46 (2.2%)	56 (5.8%)
*E. faecium* (71)	27	44
*E. gallinarum* (23)	15	8
*E. faecalis* (5)	3	2
*E. casseliflavus* (2)	1	1
*E. hirae* (1)	0	1

## Discussion

The first VRE reported from Hungary was a *vanA* positive *E. faecalis* isolated from a toe ulcer in 2000 (Ghidán et al. 2000). Since then, several publications have outlined the importance of growing prevalence of VRE infections in our country (Böröcz et al. 2005; Libisch et al. 2008). Between 2004 and 2009, we found predominantly *VanC* positive *E. gallinarum* from clinical samples at our hospital, and recently, we published the elevation of invasive and noninvasive vancomycin-resistant *E. faecium* (Franyó et al. 2018; Dombrádi et al. 2012). Up until now, there has been no information on fecal colonization rates with VRE of patients admitted to our hospital, despite the fact that carriers can serve as source of nosocomial infections.

In 2016, our microbiology laboratory showed a VRE prevalence of 3.2% (between January and December, with 6 μg/mL vancomycin containing solid screening medium used in routine culturing, unpublished data). In our study, we aimed to assess gastrointestinal VRE carriage rates in two periods in 2016 and found rates of 2.2% with the same screening medium used in routine culturing and 5.8% with enrichment broth.

Among the different geographic regions in Europe, VRE colonization rate is approximately 20% (Alevizakos et al. 2016). Although VRE-positive carriers may never develop subsequent infection, colonization can be an important sign for loss of colonization resistance against pathogens (Zhou et al. 2019). Once colonization occurs, it may persist for months to years (Nelson et al. 2014). The source of VRE according to several authors’ suggestion is contaminated food products which may serve as a reservoir from which nonhospitalized individuals can acquire VRE (Cetinkaya et al. 2000). Fortunately, the growth promoter avoparcin—which selects VRE in animals—was banned in Hungary in 1998 and in the following years, a decline in VRE rates in broiler chickens was reported (Kaszanyitzky et al. 2007). Still in 2005, 2 *vanA* VRE were isolated from slaughtered poultry (Ghidán et al. 2008).

Medical records of 93 VRE carriers were analyzed; 24.7% of the their routine culturing revealed absence of coliform bacterial normal flora at least once at the time of screening or in the previous 6 months (Table 1). This is an alarming sign of microflora imbalance which was present in one-fourth of the carriers. Twenty-two (23.7%) VRE carriers had no medical history of microbiological culturing before stool screening and among the remaining 71, only 3 were positive for VRE from other clinical specimen. However, these were all urine samples which may indicate that fecal contamination might have occurred. 8 carriers’ clinical samples yielded VRE in the following 6 months; 3 of them were the same patients mentioned above and an additional 2 were from invasive samples (blood, cannula). Interestingly, the latter were diabetic patients. Out of 93 VRE carriers, 18 were *C. difficile* positive but only 12 (66.7%) received vancomycin therapy. Seventeen VRE carriers received vancomycin but did not have a history of *C. difficile* positivity. The association between *C. difficile* positivity and VRE colonization was statistically significant (*p* = 0.0345) likewise in other studies (Özsoy and İlki 2017). The chance of VRE positivity is 2.55 times higher in CDI patients (Table 1). In vancomycin receiving patients, the chance of VRE positivity is 13.59 times higher than in the case of no vancomycin therapy. In conclusion, there is a 5.39 higher chance of becoming a VRE carrier after vancomycin therapy than after *C. difficile* infection.

Compared with previous reports, we also found malignancy as the most prevalent underlying disease (22.6%) in VRE carriers (Sohn et al. 2013; Cetinkaya et al. 2000). Alarmingly, Alevizakos et al. (2016) stated that VRE colonized patients with malignancy were 24.15 times more likely to develop bloodstream infections. In our study, infection and diabetes were nearly as common as malignancy. In an Indian teaching hospital, researchers also found that diabetes (23.7%) was the second most common risk factor in an intensive care unit (Amberpet et al. 2016). In 21.5% of our VRE carriers, there was no any other underlying disease documented in their medical history than a diagnosis related to infectious origin (Table 1). It is obvious that infections have great impact on hospital stay and the chosen antibiotic therapy. Outpatients (7/93) were also enrolled in this study so the overall hospital stay of carriers ranged from 0 to 215 days with a mean of 25 days. Prolonged hospitalization is unambiguously an important risk factor (Purohit et al. 2017; Sohn et al. 2013; Cetinkaya et al. 2000). Longer hospitalization results in extended antibiotic consumption and only 21.5% of the carriers did not receive any antibiotics. Statistical analysis of colonized and non-colonized patients revealed an increased chance of VRE colonization in the case of diabetic patients, absence of enteric normal flora, *C. difficile* positivity, longer hospital stay, and advanced age.

The anti-anaerobic drug metronidazole was the most commonly administered (47.3%) followed by cephalosporins (40.9%) and penicillins (38.7%). 31.2% of carriers received vancomycin which was the fourth most commonly administered drug together with carbapenems (Fig. 1). Additional studies have consistently shown that therapy with vancomycin, third-generation cephalosporins, and/or agents acting on anaerobes are important in the colonization process with VRE since they do not have any activity against them (Karki et al. 2012; Sohn et al. 2013; Donskey et al. 2000). In our study, we found a 13.59 times higher VRE positivity in the case of vancomycin therapy. Furthermore, there is a low-level or moderate intrinsic resistance to penicillins and carbapenems in enterococci, respectively (Top et al. 2008).

For species identification, MALDI-TOF mass spectrometry was restricted to isolates with colony morphology resembling enterococci on blood agar. This is a relatively new technique that has been recently introduced in clinical microbiology. Based on the ribosomal protein profile, it can rapidly identify a wide range of bacteria to the species level (Seng et al. 2009; Gekenidis et al. 2014). In comparison with the traditional methods applied in routine diagnosis, the advantages of MALDI-TOF include minimal sample preparation, rapid and comparable results, shorter turnaround time, and lower reagent costs (Carbonnelle et al. 2011). Altogether 102 VRE were identified to the species level with this new technique and all isolates gave results with a high score value confirming that this method is superb in the identification of bacteria. Without MALDI-TOF analysis and only relying on the blackening of the screening medium or enrichment broth, we would have interpreted the samples (with esculin-hydrolyzing organisms) as false VRE positive. So additional isolates other than enterococci would have resulted in a high rate of false positivity as stated by Ieven et al. (1999); in our case, 12.8% with solid medium and 42.2% with enrichment broth.

V*anA* strains usually predominate in the USA while in Australia, *vanB* is more frequent and in Europe, both *vanA* and *vanB* positive VRE were found (Howden et al. 2013). In our previous publication, we reported the detection of 40 *vanB* and 3 *vanA E. faecium* isolates between 2012 and 2015 from clinical samples at our hospital (Franyó et al. 2018). In 2016, VRE isolated and characterized from clinical samples were 33 *vanB* and 21 *vanA E. faecium.* This shows that not only the prevalence of VREfm is rising compared with the previous years but also the proportion of *vanA* isolates as well. VRE *E. faecalis* rates remained low (1 *vanA* and 1 *vanB* isolate, unpublished data). The present study showed that the species and genotype distribution of fecal VRE were similar to that of the clinical isolates; 71 *E. faecium* (38 *vanB*, 33 *vanA*) and 5 *E. faecalis* (1 *vanB*, *4 vanA*). Although the second most common species from stool was *E. gallinarum* (*n* = 23), it was prevalent only between 2004 and 2009 at our hospital in clinical samples (Dombrádi et al. 2012). Interestingly, the solid screen identified slightly more *E. gallinarum* than the broth but this can be due to the different time periods of screening. The proportion of *E. casseliflavus* was low (Table 3).

In one interesting case, the patient colonized with *vanA E. hirae* had previously *vanA E. faecium* isolated from his stool one month before. We assume that transfer of the *vanA* gene might have occurred between the two species, although we did not confirm this hypothesis with molecular methods. Overall, we detected 37.3% *vanA* VRE which is alarming since *vanA* can be transmitted to other nosocomial pathogens from a different genus such as *Staphylococcus aureus* (Noble et al. 1992).

Our intestinal VRE isolates were found to be sensitive to newer agents (Table 2). Only 4 out of 5 *E. faecalis* isolates were resistant to quinupristin-dalfopristin (Q/D). Although, *Enterococcus faecalis* is intrinsically resistant to Q/D because of the expression of the *lsa* gene, clinical isolates with nonsense mutations in this gene can be susceptible (Dina et al. 2003).

Prevalence of colonization with VRE reported by several studies is difficult to compare due to the differences in the target population, sample types, and detection methods using different media and/or glycopeptide concentrations (Gambarotto et al. 2000). In many hospitals, rectal swab is used for the survey of multiresistant pathogens but in the study of D’Agata et al. (2001), rectal culture failed to detect a large proportion of patients with gastrointestinal colonization. Therefore, we decided to use stool samples in our study. Several articles have documented that broth enrichment enhances the detection rates (Ieven et al. 1999; Gambarotto et al. 2000; Novicki et al. 2004). The limitation of our study was the investigation of two periods with two different methods, so direct comparison of solid medium and enrichment broth could not be carried out. Although if we focus on the enrichment method, we can observe an additional 17 *E. faecium* VRE (Table 3).

In conclusion, we showed the presence of fecal carriage of VRE at our tertiary hospital in the Eastern region of Hungary with two different methods. Stool colonization proved to be lower (2.2%) with solid screening medium but higher (5.8%) with enrichment broth compared with the prevalence of VRE infections (3.2%). Finding similar species and similar rates of *vanA* and *vanB* strains in stool VRE compared with clinical VRE might indicate the stool origin of the latter. Interestingly we found a high rate of fecal carriage of *E. gallinarum* which was present in clinical samples only until 2009 and was subsequently replaced by more resistant *vanA/vanB* strains. In view of our findings, we suggest limiting the inadequate use of antibiotics inefficient against enterococci and advise screening of risk groups such as patients with diabetes, history of recent hospitalization, or former *C. difficile* infection. Clinicians should also be aware if stool culturing indicates absence of coliform bacterial flora.

## References

[CR1] Alevizakos M, Gaitanidis A, Nasioudis D (2016). Colonization with vancomycin-resistant enterococci and risk for bloodstream infection among patients with malignancy: a systematic review and meta-analysis. Open Forum Infect Dis.

[CR2] Amberpet R, Sistla S, Parija SC (2016). Screening for intestinal colonization with vancomycin resistant enterococci and associated risk factors among patients admitted to an adult intensive care unit of a large teaching hospital. J Clin Diagn Res.

[CR3] Böröcz K, Szilágyi E, Kurcz A (2005). First vancomycin-resistant Enterococcus faecium outbreak reported in Hungary. Euro Surveill.

[CR4] Carbonnelle E, Mesquita C, Bille E (2011). MALDI-TOF mass spectrometry tools for bacterial identification in clinical microbiology laboratory. Clin Biochem.

[CR5] Cetinkaya Y, Falk P, Mayhall CG (2000). Vancomycin-resistant enterococci. Clin Microbiol Rev.

[CR6] Cheng VCC, Chen JHK, Tai JWM (2014). Decolonization of gastrointestinal carriage of vancomycin-resistant Enterococcus faecium: case series and review of literature. BMC Infect Dis.

[CR7] D’Agata EMC, Jirjis J, Gouldin C (2001). Community dissemination of vancomycin-resistant Enterococcus faecium. Am J Infect Control.

[CR8] Depardieu F, Perichon B, Courvalin P (2004). Detection of the van alphabet and identification of enterococci and staphylococci at the species level by multiplex PCR. J Clin Microbiol.

[CR9] Dina J, Malbruny B, Leclercq R (2003). Nonsense mutations in the lsa-like gene in Enterococcus faecalis isolates susceptible to lincosamides and streptogramins A. Antimicrob Agents Chemother.

[CR10] Dombrádi Z, Dobay O, Nagy K (2012). Prevalence of vanC vancomycin-resistant enterococci in the teaching hospitals of the University of Debrecen, Hungary. Microb Drug Resist.

[CR11] Donskey CJ, Chowdhry TK, Hecker MT (2000). Effect of antibiotic therapy on the density of vancomycin-resistant enterococci in the stool of colonized patients. N Engl J Med.

[CR12] European Centre for Disease Prevention and Control (2016). Annual epidemiological report 2016: healthcare-associated infections acquired in intensive care units.

[CR13] European Centre for Disease Prevention and Control (2017). Surveillance of antimicrobial resistance in Europe 2016. Annual Report of the European Antimicrobial Resistance Surveillance Network (EARS-Net).

[CR14] Franyó D, Kocsi B, Lesinszki V (2018). Characterization of clinical vancomycin-resistant Enterococcus faecium isolated in Eastern Hungary. Microb Drug Resist.

[CR15] Gambarotto K, Ploy MC, Turlure P (2000). Prevalence of vancomycin-resistant enterococci in fecal samples from hospitalized patients and nonhospitalized controls in a cattle-rearing area of France. J Clin Microbiol.

[CR16] Gekenidis MT, Studer P, Wüthrich S (2014). Beyond the matrix-assisted laser desorption ionization (MALDI) biotyping workflow: in search of microorganism-specific tryptic peptides enabling discrimination of subspecies. Appl Environ Microbiol.

[CR17] Ghidán Á, Csiszár K, Cs J (2000). PCR detection of the vanA gene in a vancomycin-resistant Enterococcus faecalis. J Antimicrob Chemother.

[CR18] Ghidán A, Dobay O, Kaszanyitzky EJ (2008). Vancomycin resistant enterococci (VRE) still persist in slaughtered poultry in Hungary 8 years after the ban on avoparcin. Acta Microbiol Immunol Hung.

[CR19] Howden BP, Holt KH, Lam MMC (2013). Genomic insights to control the emergence of vancomycin-resistant enterococci. mBio.

[CR20] Huycke MM, Sahm DF, Gilmore MS (1998). Multiple-drug resistant enterococci: the nature of the problem and an agenda for the future. Emerg Infect Dis.

[CR21] Ieven M, Vercauteren E, Descheemaeker P (1999). Comparison of direct plating and broth enrichment culture for the detection of intestinal colonization by glycopeptide-resistant enterococci among hospitalized patients. J Clin Microbiol.

[CR22] Jett BD, Huycke MM, Gilmore MS (1994). Virulence of enterococci. Clin Microbiol Rev.

[CR23] Karki S, Houston L, Land G (2012). Prevalence and risk factors for VRE colonisation in a tertiary hospital in Melbourne, Australia: a cross sectional study. Antimicrob Resist Infect Control.

[CR24] Kaszanyitzky EJ, Tenk M, Ghidán A (2007). Antimicrobial susceptibility of enterococci strains isolated from slaughter animals on the data of Hungarian resistance monitoring system from 2001 to 2004. Int J Food Microbiol.

[CR25] Leclercq R, Derlot E, Duval J (1988). Plasmid-mediated resistance to vancomycin and teicoplanin in Enterococcus faecium. N Engl J Med.

[CR26] Libisch B, Lepsanovic Z, Top J (2008). Molecular characterization of vancomycin-resistant Enterococcus spp. clinical isolates from Hungary and Serbia. Scand J Infect Dis.

[CR27] Muto CA, Jernigan JA, Ostrowsky BE (2003). SHEA guideline for preventing nosocomial transmission of multidrug-resistant strains of Staphylococcus aureus and Enterococcus. Infect Control Hosp Epidemiol.

[CR28] Nelson I, Higuita A, Huycke MM, Gilmore MS, Clewell DB, Ike Y, Shankar N (2014). Enterococcal disease, epidemiology, and implications for treatment. Enterococci: From Commensals to Leading Causes of Drug Resistant Infection.

[CR29] Noble WC, Virani Z, Cree RG (1992). Co-transfer of vancomycin and other resistance genes from Enterococcus faecalis NCTC 12201 to Staphylococcus aureus. FEMS Microbiol Lett.

[CR30] Novicki TJ, Schapiro JM, Ulness BK (2004). Convenient selective differential broth for isolation of vancomycin-resistant enterococcus from fecal material. J Clin Microbiol.

[CR31] Özsoy S, İlki A (2017). Detection of vancomycin-resistant enterococci (VRE) in stool specimens submitted for Clostridium difficile toxin testing. Braz J Microbiol.

[CR32] Purohit G, Gaind R, Dawar R (2017). Characterization of vancomycin resistant enterococci in hospitalized patients and role of gut colonization. J Clin Diagn Res.

[CR33] Seng P, Drancourt M, Gouriet F (2009). Ongoing revolution in bacteriology: routine identification of bacteria by matrix-assisted laser desorption ionization time-of-flight mass spectrometry. Clin Infect Dis.

[CR34] Sohn KM, Peck KR, Joo EJ (2013). Duration of colonization and risk factors for prolonged carriage of vancomycin-resistant enterococci after discharge from the hospital. Int J Infect Dis.

[CR35] Stosor V, Peterson LR, Postelnick M (1998). Enterococcus faecium bacteremia: does vancomycin resistance make a difference?. Arch Intern Med.

[CR36] The European Committee on Antimicrobial Susceptibility Testing. Breakpoint tables for interpretation of MICs and zone diameters. Published 2017. Version 7.1. Accessed 10 Dec 2018

[CR37] Top J, Willems R, Bonten M (2008). Emergence of CC17 Enterococcus faecium: from commensal to hospital-adapted pathogen. FEMS Immunol Med Microbiol.

[CR38] Uttley AH, Collins CH, Naidoo J (1988). Vancomycin-resistant enterococci. Lancet.

[CR39] Yean CY, Yin LS, Lalitha P (2007). A nanoplex PCR assay for the rapid detection of vancomycin and bifunctional aminoglycoside resistance genes in Enterococcus species. BMC Microbiol.

[CR40] Zhou M.J., Li J., Salmasian H., Zachariah P., Yang Y-X., Freedberg D.E. (2019). The local hospital milieu and healthcare-associated vancomycin-resistant enterococcus acquisition. Journal of Hospital Infection.

